# Hepatitis B vaccination for international travelers to Asia

**DOI:** 10.1186/s40794-016-0031-z

**Published:** 2016-08-17

**Authors:** Kittiyod Poovorawan, Ngamphol Soonthornworasiri, Patiwat Sa-angchai, Chayasin Mansanguan, Watcharapong Piyaphanee

**Affiliations:** 1 Department of Clinical Tropical Medicine, Faculty of Tropical Medicine, Mahidol University, Bangkok, Thailand; 2 Department of Tropical Hygiene, Faculty of Tropical Medicine, Mahidol University, Bangkok, Thailand

**Keywords:** Hepatitis B, Travelers, Prevalence, Vaccination, Asia, Immunization programs

## Abstract

There is a wide range in prevalence of hepatitis B virus (HBV) infection and HBV immunization programs between different regions. Hepatitis B is a vaccine preventable disease yet is still endemic in the majority of countries in Asia. Despite the decreasing global prevalence of chronic HBV infection, there is still considerable risk of HBV infection among international travelers to high endemic areas. Numbers of international travelers are expected to increase year by year; thus immunization among this cohort is a crucial preventive measure. Among international travelers to Asia, HBV immunization should be recommended for those without previous HBV vaccination who plan to travel to countries with intermediate to high prevalence of HBV, and especially for those individuals at greater risk of HBV infection; including travelers engaging in casual sex, getting a tattoo or piercing, and those having dental surgery or other medical procedures. Longer duration of travel is also associated with a greater risk of HBV infection. Travelers from low HBV prevalence countries, especially those born before implementation of universal HBV vaccination, might benefit from HBV vaccination during long-term traveling to HBV intermediate to high endemic country.

## Background

Viral hepatitis is now one of the major causes of death through communicable disease [1]. WHO estimates that 240 million people currently have chronic hepatitis B virus (HBV) infection [2]. HBV infection accounts more than 1 million deaths worldwide from cirrhosis, liver failure, and hepatocellular carcinoma [3]. Despite advances in treatment, eradication of the hepatitis B virus from patients with chronic hepatitis B is rarely achieved [4]. Prevention through vaccination is vital to control HBV infection.

Data from the GeoSentinel Surveillance Network shows that the most common vaccine preventable diseases among travelers returning home ill were enteric fever, acute viral hepatitis and influenza. Hepatitis B infection was the fourth most common after enteric fever, acute hepatitis A and influenza [5]. A study of Australian and European travelers found that approximately 30–65 % of travelers to HBV endemic countries undertook activities that potentially exposed them to HBV [6, 7]. Furthermore, less than half of the travelers (46 %) had been vaccinated against HBV [6]. The HBV vaccination rate of people travelling abroad is different in each region [8]. There are many reasons why people did not opt for pre-travel vaccinations, these include the traveler’s lack of awareness regarding the prevention of diseases during overseas travel, the limited number of healthcare vaccination facilities and that some countries have yet to approve a number of vaccines needed by travelers [9]. This review aims to explore the need for HBV vaccination among international travelers to Asia.

### International travelers to Asia

Asia is one of the major global tourist destinations, with more than 263 million international tourist arrivals in 2014 [10]. Six of the top ten most visited cities were located in Asia [11]. China received more than 55 million visits in 2014, making it the most visited country in Asia that year [10]. Most tourist countries in Asia have intermediate to high HBV prevalence. Most of these countries have implemented universal HBV vaccination. However, vaccine coverage and completeness, measured by delivery of all three doses, of HBV vaccination are still limited in some countries [12]. Furthermore, the majority of those receiving immunization tend to be the young population who were born after the initiation of WHO’s Expanded Program on Immunization (EPI) (Table 1).

**Table 1:** Prevalence of chronic hepatitis B and coverage of expanded program on HBV immunization in Asian countries receiving a high number of travelers [10, 12, 14]

Arrival Country	International traveler’s arrivals per year (2014)	Estimated prevalence of chronic hepatitis B infection^a^	Estimated HBsAg positive population	Implement of Expanded program of immunization (EPI) for HBV (Year)	Complete HBV vaccination at year 2014 (%)	Population age after EPI deployed at year 2016
China	55,622,000	5.49 %	74,601,204	2000	99	16
Malaysia	27,437,000	0.74 %	208,540	1989	96	27
Thailand	24,780,000	6.42 %	4,260,008	1992	99	24
Saudi Arabia	15,098,000	3.18 %	866,675	1990	98	26
South Korea	14,202,000	4.36 %	2,111,914	1995	99	21
Japan	13,413,000	1.02 %	1,294,431	No	No	0
Singapore	11,858,000	4.09 %	207,943	1990	97	26
Indonesia	9,435,000	1.86 %	4,468,684	1992	78	24
India	7,703,000	1.46 %	17,553,389	2004	70	12
Vietnam	7,874,000	10.79 %	9,607,438	2003	95	13
Philippines	4,833,000	4.63 %	4,326,212	1995	79	21
Cambodia	4,503,000	4.05 %	581,596	2006	97	10
Jordan	3,990,000	1.86 %	119,919	1995	98	18
Myanmar	3,081,000	3.40 %	1,765,643	2003	75	13
Laos	2,510,000	8.74 %	558,710	2003	88	13

Estimated at year 2015 based on data on prevalence of chronic HBV infection published between Jan 1, 1965, and Oct 23, 2013^a^

A large proportion of international travelers to Asia depart from Europe and North America [13]. Most countries in those regions are classified as low HBV prevalence [14]. Therefore many countries do not have universal HBV vaccination [12]. Overall prevalence of hepatitis B infection and coverage of the Expanded Program on HBV Immunization in selected countries with high international traveler’s departures to Asia is summarized in Table 2.

**Table 2:** Prevalence of CHB and coverage of expanded program on HBV immunization in international traveler’s to Asia departure countries outside Asia [12–14]

Region	Country	International traveler’s departures per year (2013)	Estimated prevalence of chronic hepatitis B infection^a^	Estimated HBsAg positive population	Start of Expanded Program of Immunization (EPI) for HBV (Year)	Complete HBV vaccination (%)	Number of years since EPI deployed, at year 2016
N. America	USA	61,569,000	0.27 %	843,724	1993	90	23
	Canada	32,977,000	0.76 %	260,865	2003	75	13
S. America	Mexico	15,911,000	0.20 %	237,858	2000	84	16
	Argentina	7,544,000	0.77 %	312,806	2002	94	14
Europe	United Kingdom	58,510,000	0.01 %	3,300	Not started	N/A	N/A
	Russia	54,069,000	2.73 %	3,926,499	2001	97	15
	Italy	27,798,000	2.52 %	1,522,546	1991	94	25
	France	26,243,000	0.26 %	165,728	1998	82	18
	Ukraine	23,761,000	1.45 %	666,280	2000	46	16
	Netherlands	18,094,000	0.40 %	67,009	2013	95	2
	Hungary	15,997,000	0.53 %	53,301	Not started	N/A	N/A
	Sweden	15,917,000	0.59 %	55,606	2011	42	5
	Spain	11,246,000	0.34 %	158,287	1996	96	20
Oceania	Australia	8,768,000	0.37 %	83,121	2001	91	15
	New Zealand	2,193,000	4.11 %	179,357	1992	93	24
Africa	South Africa	5,168,000	6.70 %	3,445,477	1997	74	19
	Uganda	378,000	9.19 %	3,123,886	2002	78	14

^a^ Estimated at year 2015 based on data on prevalence of chronic HBV infection published between Jan 1, 1965, and Oct 23, 2013

### Risk of HBV infection during travel

HBV transmission routes in travelers are through percutaneous or mucosal exposure of HBV‐infected blood or bodily fluids including saliva or semen; via sexual contact, contaminated blood products, contaminated medical equipment, and via sharing needles and injecting apparatus [15]. In people using needles contaminated with HBV, the risk of developing infection is approximately 30 % [16]. These routes are different from the usual routes of transmission in high prevalence areas where vertical transmission, when an infected mother transmits the infection to her offspring, is most common [17].

Travelers such as expatriates, those visiting friends and relatives, and travelers engaging in casual sex, dental surgery, and medical procedures may be at greater risk of HBV infection [6]. Younger travelers and those travelers with a longer duration of travel are also at greater risk of HBV infection [18, 19]. A study of international backpackers in Thailand, found that 25 % of those travelers had had casual sex while travelling and almost half did not always use condom [20]. A study of Dutch travelers to tropical and sub-tropical destinations found that the risk of infection from unprotected sex increased with the number of partners and the incidence of unprotected sex was higher among single travelers [21]. A study by Leggat PA, et al., showed that about half of Australian travelers had participated in at least one activity with a HBV risk during their last overseas trip to Southeast or East Asia [22]. Non-immune travelers traveling to high HBV prevalence countries might be at risk of HBV infection due to the many potential accidental and uncontrollable exposures during travel.

### Prevalence of HBV infection in Asia

**Fig. 1:**
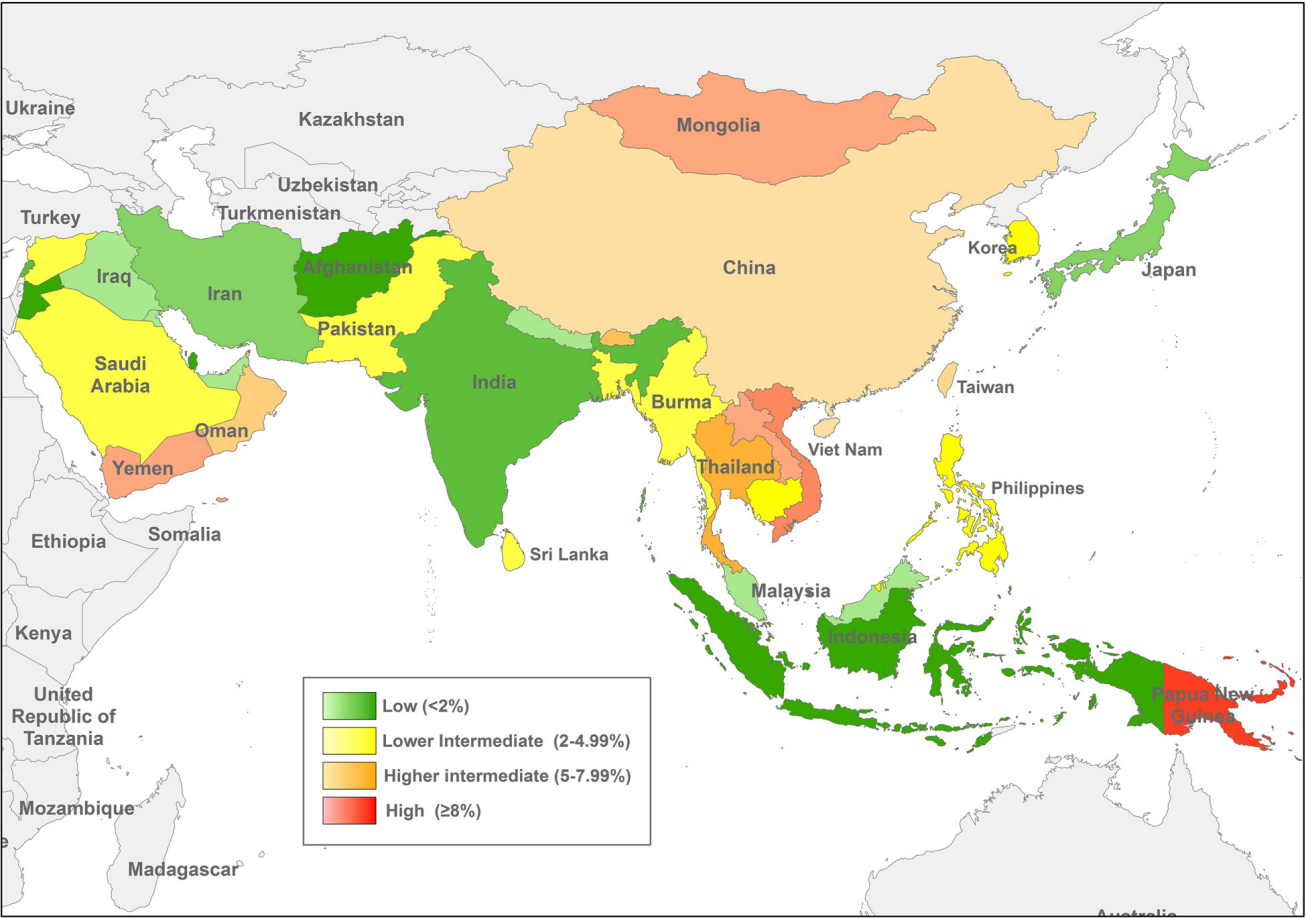
Estimated prevalence of chronic hepatitis B infection in Asia at 2015

HBsAg seroprevalence is estimated to be around 3.61 % worldwide. The seroprevalence varies in different regions, with the lowest rates in North America and the highest in Africa. Overall prevalence of HBV infection in Asia is estimated to be 5.26 %, with rates varying between countries [14]. Estimated prevalence of chronic hepatitis B infection in Asian countries receiving a high number of travelers is summarized in Table 1. HBV infection is highly endemic in Southeast Asia and China with a rate of chronic infection of 7–10 % among the general population in these areas [23, 24]. Prevalence of HBV infections are classified as low (<2 %), low intermediate (2–4.99 %), high intermediate (5–7.99 %) and high (≥8 %). Estimates based on published data of prevalence of HBV infections from 1965 to 2013 show many countries in Asia to be intermediate to high endemic (Fig. 1) [14]. Based on recent data, Mongolia, Laos, Vietnam and Papua New Guinea are classified with having high prevalence of chronic hepatitis B infection [14].

A study by Posuwan N, et al. showed that the prevalence on HBsAg positive subjects in Thailand decreased from 5–6 % to less than 1 % by 2014, after the implementation of EPI in 1992 [17]. Studies from Luo Z et al., Mohammadi Z, et al. and Kim H, et al. have also shown the impact of EPI through documenting the decreasing prevalence of HBV infection in China, Iran and Korea [23, 25, 26].

**Fig. 2:**
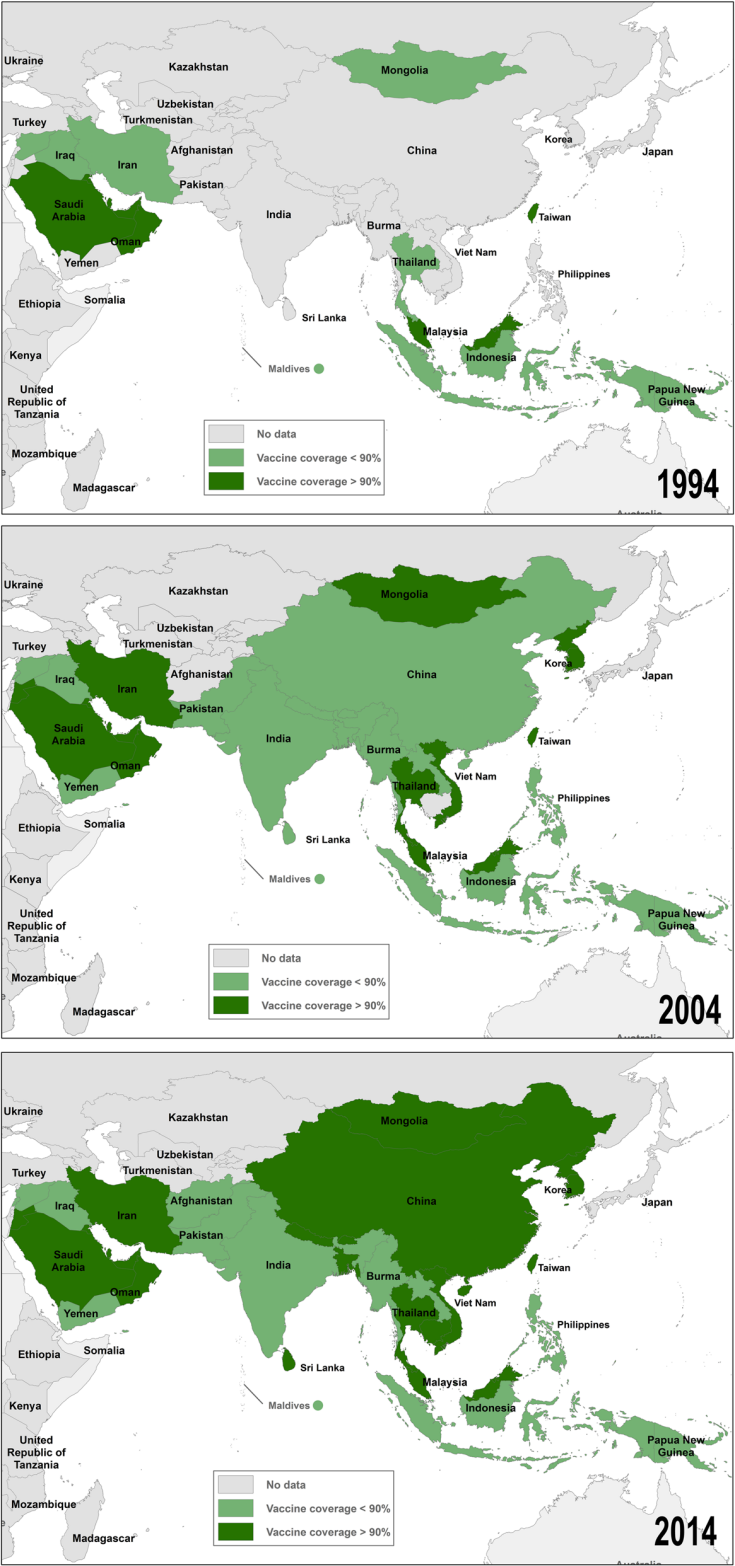
Estimated coverage of HBV vaccination in Asia in 1994, 2004, 2014

Universal HBV vaccination was recommended by World Health Organization (WHO) in 1997 [27]. In the 20 years that followed, almost all countries in Asia incorporated HBV vaccination into their national infant immunization programs [12]. In 2014, WHO and UNICEF estimated 62 % of countries in Asia had achieved more than 90 % coverage of completed (three doses) HBV vaccination (Fig. 2) [12, 28]. The prevalence of HBV infection and the risk to travelers are likely to decrease as universal vaccination of infants is progressively implemented [29].

Universal HBV vaccination decreases HBsAg seroprevalence in young age groups and vaccine induced protection from HBV infection in the young population after implementation of universal HBV vaccination is moderately high (68.5 %) [17, 30].

Vaccine effectiveness and EPI has led to a strong reduction in HBsAg prevalence in Southeast Asia in the youngest age group (0–14 years) where prevalence levels were 1.2–1.4 % in 2005. In contrast, prevalence in Southeast Asian adults was still high intermediate and this age group is the most likely to interact with travelers [31].

### HBV Vaccination for travelers

Current commercially available hepatitis B vaccines are the recombinant Hepatitis B vaccine (Engerix-B®, GlaxoSmithKline and Recombivax HB®, Merck & Co., Inc.) and the combined hepatitis A and B vaccine (Twinrix®, GlaxoSmithKline). The complete hepatitis B vaccination needs 3 doses of vaccine. The usual schedule of the three intramuscular injections is to have the second and third administered 1 and 6 months after the first. An accelerated schedule (doses on days 0, 7, 21, and then a post-travel dose at 12 months) may be used if there is insufficient time for pre‐travel vaccination [32].

HBV prevalence varies between countries, and therefore the number of people acquiring protective immunity from a previous HBV infection also varies. Recommendation of HBV vaccination should be based on likelihood of infection during travel and evidence of previous immunization from either vaccination or recovery from previous infection. In those travelers without evidence of previous HBV immunity, HBV vaccination is recommended in those with HBV exposure risks and travelling to HBV endemic country.

The US Center of Disease Control and Prevention (CDC) recommends HBV vaccination to all unvaccinated people traveling to areas with intermediate to high prevalence of chronic hepatitis B and suggests it should be considered for all international travelers, regardless of destination, depending on the traveler’s potential risk exposure. High risk activities include unprotected sex with a new partner, getting a tattoo or piercing, or having any medical procedures [33].

Despite the CDC recommendation, a study by Connor BA, et al. showed that only 19 % of all American travelers and 30 % of American travelers planning high risk activities had received a completed hepatitis B vaccination before departure [18]. This information is consistent with data from Europe that only 15 % international travelers to HBV endemic countries receive a completed hepatitis B vaccination before travel [34].

**Fig. 3:**
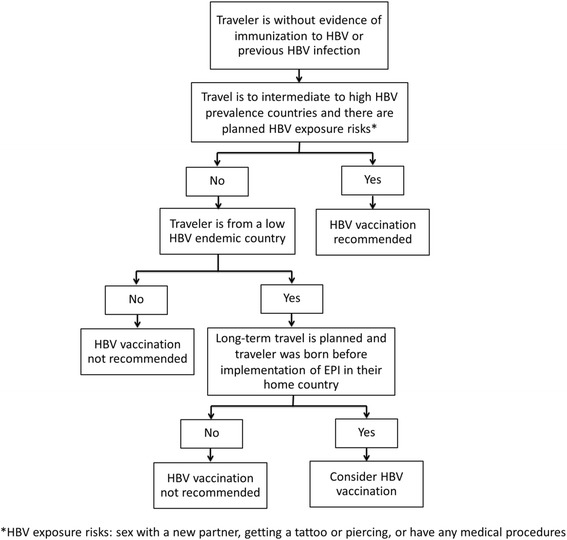
Decision Tree for Hepatitis B vaccination for international travelers to Asia

In travelers from low endemic countries and who were born before EPI, the chance of immunity to HBV is very low [30, 35]. Currently there are no recommendations for HBV serologic screening of international travelers. Due to the high numbers of people it is impractical to screen all international travelers and only 3.4–3.9 % of the population in low endemic countries will have serologic evidence of prior infection [30, 35]. Immunization of those individuals should be considered, especially if long-term travel is planned to countries with intermediate to high prevalence of HBV (Fig. 3).

## Conclusions

Hepatitis B is still endemic in the majority of countries in Asia. HBV infection during travel might occur in those without HBV immunity traveling in an endemic country. This article summarized the updated data that should influence a traveler’s decision on whether to get the HBV vaccination before travel to Asia. Vaccination is still the best preventive measure and should be considered by those at risk of HBV infection during travel.

## Abbreviations

EPI: Expanded program of immunization
HBsAg: Hepatitis B surface antigen
HBV: Hepatitis B virus
